# Hepatitis B and C in individuals with a history of antipsychotic medication use: A population-based evaluation

**DOI:** 10.1371/journal.pone.0284323

**Published:** 2023-04-14

**Authors:** Amnah Awan, Sharara Shakik, Hailey R. Banack, David N. Fisman, Alison E. Simmons

**Affiliations:** Division of Epidemiology, Dalla Lana School of Public Health, University of Toronto, Toronto, Ontario, Canada; George Washington University, UNITED STATES

## Abstract

**Background:**

A better understanding of links between mental illness and risk of bloodborne infectious disease could inform preventive and therapeutic strategies in individuals with mental illness.

**Methods:**

We performed a cross-sectional study using the National Health and Nutrition Examination Survey (NHANES) to estimate the seroprevalence of hepatitis B and C in individuals with and without a prior prescription for antipsychotic medications, and to determine whether differences in seroprevalence could be explained by differential distribution in known infection risk factors. Multivariable logistic regression models were used to examine the association between receipt of antipsychotic medication and HBV and HCV seropositivity.

**Results:**

Those who had HBV core antibody had 1.64 (95% CI: 0.89, 3.02) times the odds and those with HCV antibody (anti-HCV) had 3.48 (95% CI: 1.71, 7.09) times the odds of having a prescription for at least one antipsychotic medication compared to those who did not have HBV core antibody or HCV antibody, respectively. While prior antipsychotic receipt was a potent risk marker for HCV seropositivity, risk was explained by adjusting for known bloodborne infection risk factors (adjusted ORs 1.01 [95% CI: 0.50, 2.02] and 1.38 [95% CI: 0.44, 4.36] for HBV and HCV, respectively).

**Conclusions:**

Prior receipt of antipsychotic medications is a strong predictor of HCV (and to a lesser extent HBV) seropositivity. Treatment with antipsychotic medications should be considered as additional risk markers for individuals who may benefit from targeted prevention, screening, and harm reduction interventions for HCV.

## Introduction

Bloodborne viral infections, including hepatitis B and C viruses (HBV and HCV respectively) are a major cause of morbidity and premature death globally [1, 2]. There have been dedicated, international efforts to end epidemics caused by these bloodborne viruses over the last few decades through immunization, antiviral treatment, prevention of mother-to-infant transmission, and effective screening of blood and blood products, but transmission risk persists, often in association with injection drug use and sexual behaviors that increase the risk of disease transmission [3].

Individuals with major mental illness are at increased risk of a variety of adverse health outcomes [4], and data suggest that those with major mental illness may be at increased risk of bloodborne infection, though this issue has received limited study in recent years [5–9]. In work published in 2001, Rosenberg and colleagues noted that individuals receiving care for major mental illness in four U.S. states had seroprevalences of HBV infection and HCV infection that were 5- and 11-fold higher, respectively, than the general population [5].

The association between major mental illness and increased risk of bloodborne infection is likely to be multifactorial. Individuals living with severe mental illness are at increased risk of acquiring bloodborne infectious diseases due to higher rates of engagement in behaviors including injection drug use, sex with multiple partners, and sex work [9, 10]. The impact of such infections may previously have been increased in those with mental illness, as early treatments (e.g., interferons for HCV infection) were considered relatively contraindicated in individuals with some mental illnesses [11]. Additionally, current antiviral drugs may interact with drugs used to treat mental illness [12, 13].

Given the evolution in the epidemiology of these diseases in recent years and the changing availability and effectiveness of preventive measures and treatment since the publication of Rosenberg’s work in 2001 [5], we sought to re-assess the association between major mental illness and risk of HBV and HCV. Furthermore, given that existing research on this topic in the United States has often been performed in populations institutionalized with major mental illness, or specific sub-populations (e.g., individuals experiencing homelessness), we had concerns about potential biases and the generalizability of existing research [9]. As such, we sought to evaluate the association between major mental illness and risk of HBV and HCV using the National Health and Nutrition Examination Survey, a population representative sample of non-institutionalized civilian population in the United States [14]. As the use of antipsychotic medications, which are primarily used for the control of psychotic symptoms, was recorded in this dataset, we used receipt of medications in the preceding 30 days from this class as a surrogate measure for major mental illness.

Our objectives were to (i) construct age-seroprevalence curves for HBV and HCV; and (ii) evaluate whether associations between HBV and HCV infection risk and receipt of antipsychotic medication were present, and whether they persisted after adjustment for bloodborne infection risk factors.

## Methods

### Data

We conducted a secondary analysis of data obtained from the National Health and Nutrition Examination Survey (NHANES) to determine the association between antipsychotic medication use and risk of bloodborne infection. NHANES is a survey conducted by the National Center for Health Statistics in a 2-year cycle that uses a stratified, multistage probability sampling design to represent the non-institutionalized, civilian population of the United States [14]. NHANES includes self-reported demographic, socioeconomic, and health-related information from a questionnaire, as well as medical, physiological, and laboratory measurements (more information on NHANES can be found at https://wwwn.cdc.gov/nchs/nhanes/). For this study, we combined publicly available data from NHANES collected between 2005 and 2014 (cycles 4–8).

### Measures

The exposure was antipsychotic use, as defined by at least one prescription antipsychotic medication (i.e., aripiprazole, asenapine, brexpiprazole, cariprazine, chlorpromazine, clozapine, fluphenazine, haloperidol, iloperidone, loxapine, lurasidone, olanzapine, paliperidone, perphenazine, prochlorperazine, quetiapine, risperidone, trifluoperazine, ziprasidone) taken in the past 30 days [15]. The outcomes were lifetime history of HBV or HCV infection, as defined by the positive identification of HBV core antibody or HCV antibody (anti-HCV).

Risk factors included in our models were age, sex, race, immigrant status, marital status, education, income, history of a blood transfusion, alcohol use, injection drug use, and history of a sexually transmitted infection (STI) [5, 16–18]. The selected sociodemographic factors represent structural factors that influence access to health care and health information (e.g., sex, race, immigrant status, marital status, education, and income) and risk factors for bloodborne infection (e.g., age, immigrant status, history of blood transfusion, alcohol use, injection drug use, and history of a sexually transmitted infection). A directed acyclic graph (DAG) in S1 Fig illustrates the relationship between antipsychotic use, bloodborne infection, and bloodborne infection risk factors. Age was treated as a continuous variable in our model. Sex was a binary variable: male and female. Race and ethnicity were categorized into four groups: non-Hispanic White, non-Hispanic Black, Hispanic, and all other races. Immigrant status was a binary variable based on if the respondent was born in the United States or born in a country other than the United States. Marital status was categorized into three groups: 1. married or cohabitating, 2. never married, and 3. widowed, divorced, or separated. Income was measured as the ratio of family income to the federal poverty line, with 0.00 representing no income and 5.00 representing 500% of the federal poverty line or higher. History of a blood transfusion was a binary variable. Alcohol use was binary and defined as at least one occasion in the past 12 months where at least four drinks (females) or five drinks (males) were consumed in a single day. Injection drug use was binary and included any respondent who had used a needle to inject an illicit substance at least once in their life. History of a sexually transmitted infection was binary and was defined as self-reported gonorrhea or chlamydia infection in the respondents’ lifetime.

### Analysis

**Fig 1:**
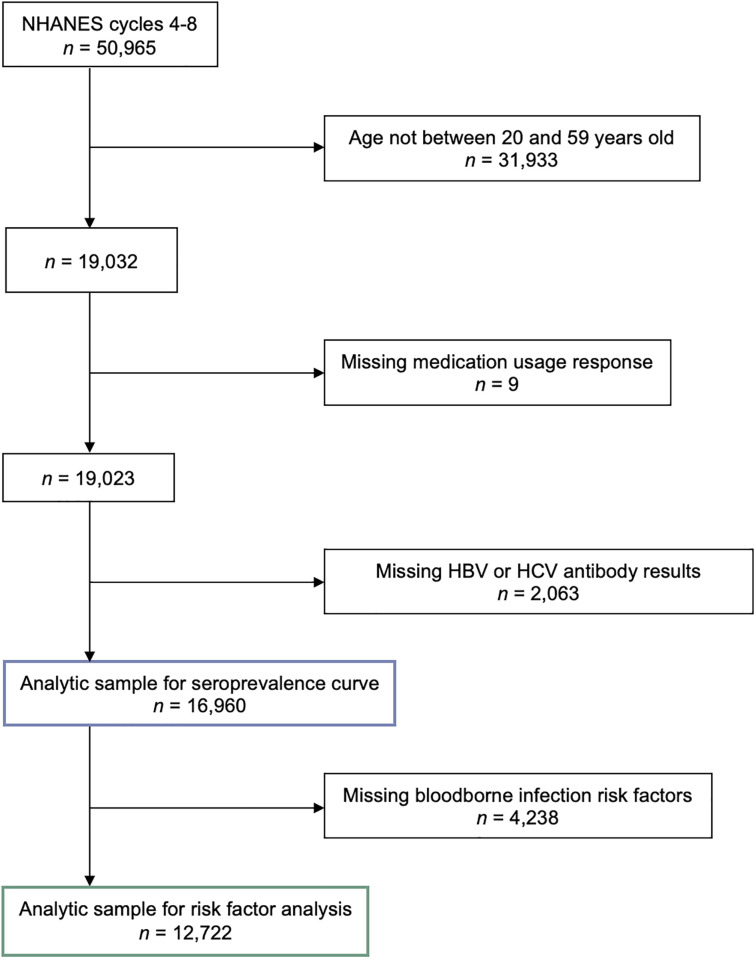
Determination of analytic sample from the 2005–2014 National Health and Nutrition Examination Surveys (NHANES).

Only NHANES respondents between ages 20 to 59 were included because not all age groups were asked the questions used in our analysis (Fig 1). In our HBV and HCV infection risk factor analysis, we also excluded respondents who were missing information on bloodborne infection risk factors. We calculated bivariate statistics to describe our analytic sample by antipsychotic use. Pearson’s chi-square tests were used for categorical responses and unpaired t-tests were used for continuous responses.

We used logistic regression to quantify the independent association between antipsychotic use and HBV antibody, and antipsychotic use and HCV antibody. Unadjusted logistic regression was just to determine the crude association between antipsychotic use, and antibodies to HBV and HCV. Subsequently, multivariable logistic regression was used to quantify the adjusted association between antipsychotic use and antibodies to each bloodborne hepatitis with adjustment for age, sex, race, immigrant status, marital status, education, income, history of a blood transfusion, alcohol use, injection drug use, and history of a sexually transmitted infection.

We used multinomial logistic regression in a secondary analysis to quantify the impact of antipsychotic use on hepatitis seroprevalence in four categories: no HBV core antibody and no HCV antibody; HBV core antibody only; HCV antibody only; and both HBV core antibody and HCV antibody.

Survey weights and design-based variance estimation methods were used in alignment with NHANES methodology [14, 19]. Analyses were conducted in Stata Statistical Software Release 17.0 (StataCorp LP, College Station, Texas) and R Version 4.2.1 (R Core Team, Vienna, Austria).

## Results

Of the 16,960 NHANES respondents included in our seroprevalence curve, 264 (1.4%) had a prescription for at least one antipsychotic medication in the month prior to their interview (S1 Table).

Of the 12,722 NHANES respondents included in our bloodborne infection risk factor analysis, 163 (1.1%) had a prescription for at least one antipsychotic medication in the month prior to their interview (Table 1). Individuals who had antipsychotic medication use had a higher prevalence of HBV antibody (5.9% compared to 3.7% [p = 0.11]) and HCV antibody (5.3% compared to 1.6% [p<0.001]) compared to those who had no antipsychotic medication use. Those with antipsychotic medication use were older (p<0.05), more likely to be born in the United States (p<0.01), never married (p<0.001), lower income (p<0.001), more likely to have received a blood transfusion (<0.05), more likely to report injection drug use (p<0.001), and more likely to have been diagnosed with a sexually transmitted infection (p<0.05). There were no significant differences in antipsychotic medication use by sex, race, education, and alcohol use.

**Table 1:** Distribution of hepatitis B and C seroprevalence, sociodemographic, and behavioral characteristics by antipsychotic medication use from the 2005–2014 National Health and Nutrition Examination Survey (*n* = 12,722).

Characteristic	Antipsychotic use		No Antipsychotic Use		*p* ^a^
	(*n* = 163)		(*n* = 12,559)		
	Wtd. %	(Unwtd. *n*)	Wtd. %	(Unwtd. *n*)	
Hepatitis B core antibody, % (*n*)					0.11
HBV positive	5.9%	(16)	3.7%	(637)	
HBV negative	94.1%	(147)	96.3%	(11,922)	
Hepatitis C virus antibody, % (*n*)					<0.001
HCV positive	5.3%	(13)	1.6%	(216)	
HCV negative	94.7%	(150)	98.4%	(12,343)	
Age, *M* [95% CI]	41.6	[39.4, 43.8]	39.1	[38.7, 39.5]	<0.05
Sex, % (*n*)					0.31
Male	54.1%	(82)	49.2%	(6,057)	
Female	45.9%	(81)	50.8%	(6,502)	
Race, % (*n*)					0.08
Non-Hispanic White	72.5%	(93)	67.9%	(5,548)	
Non-Hispanic Black	13.7%	(35)	11.1%	(2,609)	
Hispanic	8.4%	(24)	14.5%	(3,229)	
All other races	5.3%	(11)	6.5%	(1,173)	
Immigrant status, % (*n*)					<0.01
Born in United States	93.0%	(142)	83.4%	(9,301)	
Born outside United States	7.0%	(21)	16.6%	(3,258)	
Marital status, % (*n*)					<0.001
Married or cohabitating	46.5%	(65)	64.0%	(7,657)	
Never married	38.0%	(65)	22.5%	(3,096)	
Widowed, divorced, or separated	15.5%	(33)	13.5%	(1,806)	
Education, % (*n*)					0.06
College	54.7%	(77)	65.0%	(7,288)	
High school graduate	26.1%	(41)	21.2%	(2,725)	
Less than high school	19.2%	(45)	13.8%	(2,546)	
Income, *M* [95% CI]^b^	2.26	[1.95, 2.58]	3.11	[3.02, 3.19]	<0.001
Blood transfusion, % (*n*)					<0.05
Yes	11.4%	(25)	6.3%	(830)	
No	88.7%	(138)	93.7%	(11,729)	
Alcohol use, % (*n*)					0.33
Yes	36.4%	(48)	41.9%	(4,864)	
No	63.6%	(115)	58.1%	(7,695)	
Injection drug use, % (*n*)					<0.001
Yes	9.2%	(12)	2.1%	(253)	
No	90.8%	(151)	97.9%	(12,306)	
Sexually transmitted infection, % (*n*)					<0.05
Yes	3.2%	(6)	1.0%	(156)	
No	96.8%	(157)	99.0%	(12,403)	

*Notes*: Wtd. = weighted; Unwtd. = unweighted; *M* = mean; CI = confidence interval;

^*a*^Pearson’s chi-square for categorial and *t* test for continuous predictors;

^*b*^Income is measured by the ratio of family income to the federal poverty lines, with values from 0 to 5. A value of “0” represents no income and a value of “5” represents an income greater than or equal to 500% of the federal poverty line.

**Fig 2:**
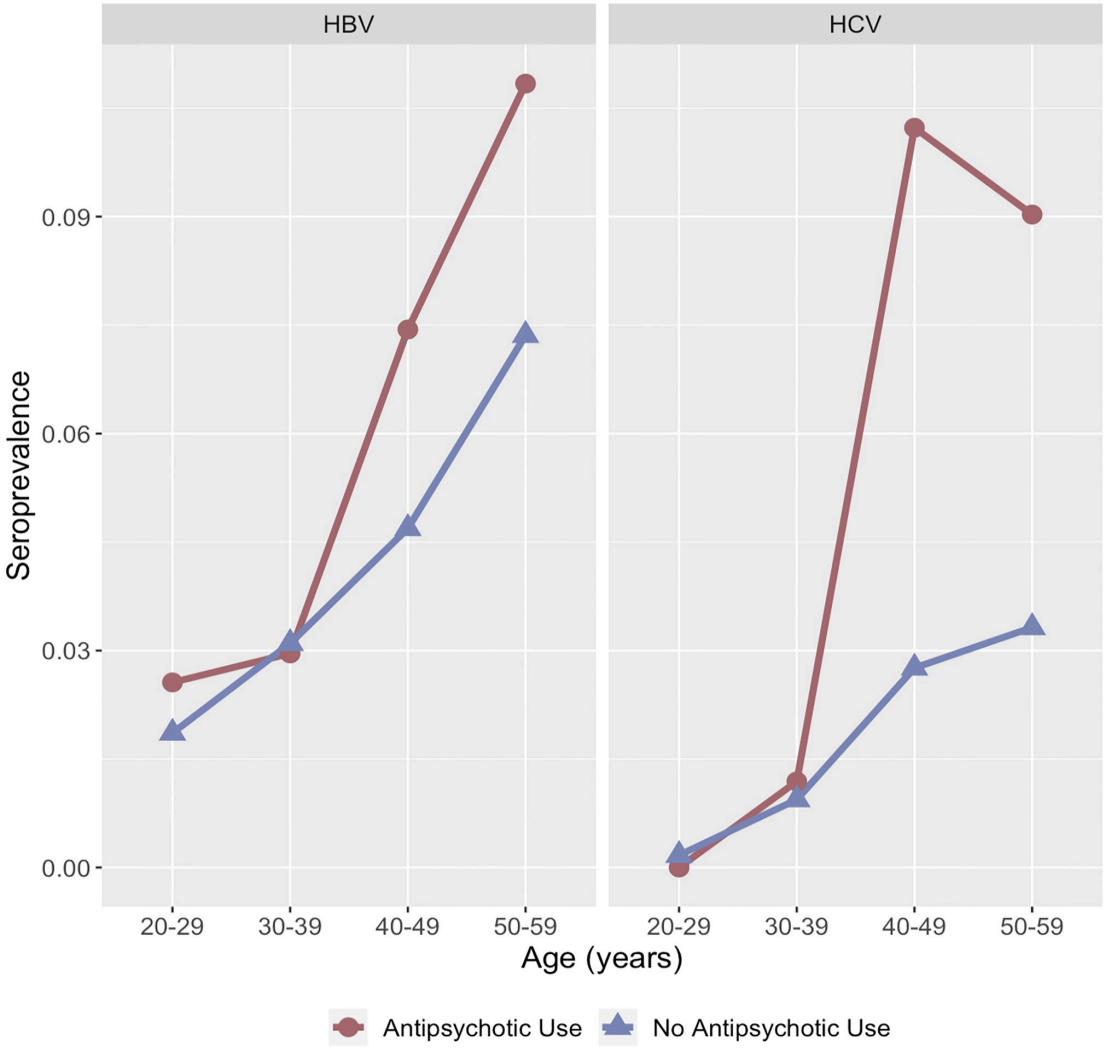
Population weighted seroprevalence of hepatitis B virus (HBV) and hepatitis C virus (HCV) by age group among respondents to the 2005–2014 National Health and Nutrition Examination Survey (*n* = 16,960).

The seroprevalence of HBV and HCV differed by antipsychotic medication use and across age groups (Fig 2). Among both groups, the seroprevalence of HBV and HCV was low between the ages of 20 to 39. The seroprevalence among the respondents with no antipsychotic medication use increased at a constant rate across age groups. In contrast, the seroprevalence among respondents with antipsychotic medication use rose between ages 30 to 39, and ages 40 to 49.

In our unadjusted model, those who had HBV antibody had 1.64 (95% CI: 0.89, 3.02) times the odds of having a prescription for at least one antipsychotic medication compared to those who did not have HBV antibody (Table 2). After adjustment for age, sex, race, immigrant status, marital status, education, income, history of a blood transfusion, alcohol use, injection drug use, history of a sexually transmitted infection, there was no significant association (OR: 1.01 [95% CI: 0.50, 2.02]) between antipsychotic medication use and HBV antibody. In our second unadjusted model, those who had HCV antibody had 3.48 (95% CI: 1.71, 7.09) times the odds of having a prescription for at least one antipsychotic medication compared to those who did not have HCV antibody (Table 3). After adjustment for age, sex, race, immigrant status, marital status, education, income, history of a blood transfusion, alcohol use, injection drug use, and history of a sexually transmitted infection, there was no significant association (OR: 1.38 [95% CI: 0.44, 4.36]) between antipsychotic medication use and HCV antibody.

**Table 2:** Unadjusted and adjusted prevalence odds ratios, with 95% confidence intervals (CI), of the association between antipsychotic medication use and antibody to hepatitis B virus (HBV) among respondents to the 2005–2014 National Health and Nutrition Examination Survey (*n* = 12,722).

Characteristic	Unadjusted			Adjusted		
	OR	[95% CI]	*p*	aOR	[95% CI]	*p*
Antipsychotic Use						
Yes	1.64	[0.89, 3.02]	0.11	1.01	[0.50, 2.02]	0.98
No	1.00	Ref.		1.00	Ref.	
Age, *M* [95% CI]				1.06	[1.05, 1.07]	<0.001
Sex, % (*n*)						
Male				1.00	Ref.	
Female				0.73	[0.60, 0.89]	<0.05
Race, % (*n*)						
Non-Hispanic White				1.00	Ref.	
Non-Hispanic Black				4.28	[3.29, 5.55]	<0.001
Hispanic				0.81	[0.51, 1.30]	0.38
All other races				5.56	[3.99, 7.76]	<0.001
Immigrant status, % (*n*)						
Born in United States				1.00	Ref.	
Born outside United States				3.03	[2.26, 4.07]	<0.001
Marital status, % (*n*)						
Married or cohabitating				1.00	Ref.	
Never married				1.31	[0.96, 1.80]	0.09
Widowed, divorced, or separated				1.11	[0.80, 1.53]	0.52
Education, % (*n*)						
College				1.00	Ref.	
High school graduate				1.07	[0.78, 1.47]	0.68
Less than high school				1.05	[0.77, 1.43]	0.75
Income, *M* [95% CI]				0.85	[0.78, 0.92]	<0.001
Blood transfusion, % (*n*)						
Yes				1.20	[0.83, 1.71]	0.33
No				1.00	Ref.	
Alcohol use, % (*n*)						
Yes				0.99	[0.78, 1.27]	0.97
No				1.00	Ref.	
Injection drug use, % (*n*)						
Yes				6.14	[3.98, 9.46]	<0.001
No				1.00	Ref.	
Sexually transmitted infection, % (*n*)						
Yes				2.19	[1.06, 4.52]	<0.05
No				1.00	Ref.	

*Notes*: OR = bivariate prevalence odds ratio; aOR = multivariable prevalence odds ratio; Ref. = reference.

**Table 3:** Unadjusted and adjusted prevalence odds ratios, with 95% confidence intervals (CI), of the association between antipsychotic medication use and antibody to hepatitis C virus (HCV) among respondents to the 2005–2014 National Health and Nutrition Examination Survey (*n* = 12,722).

Characteristic	Unadjusted			Adjusted		
	OR	[95% CI]	*p*	aOR	[95% CI]	*p*
Antipsychotic Use						
Yes	3.48	[1.71, 7.09]	<0.01	1.38	[0.44, 4.36]	0.58
No	1.00	Ref.		1.00	Ref.	
Age, *M* [95% CI]				1.10	[1.08, 1.12]	<0.001
Sex, % (*n*)						
Male				1.00	Ref.	
Female				0.63	[0.36, 1.10]	0.10
Race, % (*n*)						
Non-Hispanic White				1.00	Ref.	
Non-Hispanic Black				1.24	[0.84, 1.81]	0.27
Hispanic				1.18	[0.60, 2.33]	0.63
All other races				0.95	[0.41, 2.20]	0.90
Immigrant status, % (*n*)						
Born in United States				1.00	Ref.	
Born outside United States				0.41	[0.15, 1.08]	0.07
Marital status, % (*n*)						
Married or cohabitating				1.00	Ref.	
Never married				1.14	[0.68, 1.95]	0.61
Widowed, divorced, or separated				0.87	[0.51, 1.49]	0.61
Education, % (*n*)						
College				1.00	Ref.	
High school graduate				1.46	[0.79, 2.70]	0.22
Less than high school				1.81	[1.02, 3.23]	<0.05
Income, *M* [95% CI]				0.67	[0.57, 0.79]	<0.001
Blood transfusion, % (*n*)						
Yes				1.91	[1.17, 3.11]	<0.05
No				1.00	Ref.	
Alcohol use, % (*n*)						
Yes				1.58	[1.03, 2.43]	<0.05
No				1.00	Ref.	
Injection drug use, % (*n*)						
Yes				33.09	[18.95, 57.76]	<0.001
No				1.00	Ref.	
Sexually transmitted infection, % (*n*)						
Yes				0.24	[0.03, 1.75]	0.16
No				1.00	Ref.	

*Notes*: OR = bivariate prevalence odds ratio; aOR = multivariable prevalence odds ratio; Ref. = reference.

## Discussion

We demonstrated that individuals with major mental illness have an inflated prevalence of HBV antibody and HCV antibody in the general United States adult population. This disparity was most pronounced among individuals over age 40. The association between major mental illness and bloodborne infections did not persist after adjustment for known HBV and HCV infection risk factors.

Our results align with findings from a meta-analysis and systematic review by Hughes and colleagues [9]. The prevalence of bloodborne infection (i.e., HIV) was consistently higher among people with major mental illness, however, the authors state that the association is likely confounded by socioeconomic status, drug and alcohol use, sex, and ethnicity. After we controlled for these factors, in addition to other risk factors, we found that major mental illness is not an isolated risk factor for bloodborne infections. Our estimates of HBV and HCV seroprevalence are slightly lower than estimates from a 2001 study among individuals undergoing inpatient or outpatient treatment for severe mental illness [5]. However, our study population included members of the general United States population, and we used antipsychotic medication prescription as a surrogate measure of severe mental illness.

To our knowledge, no population-based studies have examined the association between major mental illness and bloodborne infections in the United States [9, 20]. In a Swedish population-based study, those with severe mental illness had 2.3 times higher odds of HBV infection and 6.2 times higher odds of HCV infection compared to those without severe mental illness [20]. In our study, we included additional risk factors for bloodborne infection including history of a blood transfusion, alcohol use, a specific measure of injection drug use, and sexual behaviour. Furthermore, our outcome was HBV and HCV seroprevalence, an aggregate measure of current and previous infection.

Our findings have important practical implications. For example, in Canada, universal screening for hepatitis C virus infection is not recommended. However, screening is recommended for individuals with a number of risk factors, including a history of injection drug use, prior incarceration, or receipt of blood transfusion prior to 1992 [21]. However, mental health history is not currently considered as a factor that should motivate clinicians to screen for HCV. Given the potential health benefits associated with the treatment of chronic HCV infection and the stigmatized nature of risk factors such as injection drug use and prior mental health challenges, the review of prior prescriptions may be a useful additional clinical cue to screening.

Our study had both strengths and limitations. We used a population representative sample of the non-institutionalized, civilian adult population of the United States to examine the association between major mental illness and bloodborne infections. Additionally, we were able to incorporate a variety of risk factors for bloodborne infections to isolate our association of interest. We were also able to examine age-specific seropositivity of HBV and HCV among our study population. However, NHANES is a cross-sectional survey which prevents us from establishing temporality between major mental illness and bloodborne infections. Additionally, HCV antibody testing was not included in the 2015–2016 NHANES (cycle 9), thus we only included data up to 2014. Non-differential exposure misclassification may result from self-reported prescription use in the prior month as our measure of antipsychotic medication use which would result in underestimated prevalence odds ratios [22]. We assumed that any exposure misclassification was non-differential because reporting of prescription use occurred prior to the assessment of bloodborne infection status; and it is unlikely that individuals with HBV or HCV would differentially report prescription medication use. We were also unable to assess lifetime prescription drug use which may lead to additional non-differential exposure misclassification. We focused on antipsychotic drugs, as they are used to treat major mental illness with some specificity [23]. Additional psychotropic drugs were not considered because they are commonly used to treat non-psychiatric conditions [24]. Finally, responses to NHANES questions on sociodemographic factors, alcohol and drug use, and sexual behaviour are self-reported, and some degree of misclassification is likely.

Our findings indicate that individuals with at least one antipsychotic prescription have an increased seroprevalence of HBV and HCV compared to individuals without an antipsychotic prescription in the non-institutionalized, civilian adult population of the United States. Bloodborne infection prevention (i.e., harm reduction education and access), screening, and treatment should be integrated into mental health care for people with major mental illness and those with those with prescriptions for antipsychotic drugs [25].

## Supporting information

S1 Fig Directed acyclic graph (DAG) illustrating the relationship between antipsychotic medication use and hepatitis B and C. (TIF)

S1 Table Distribution of hepatitis B core antibody and hepatitis C antibody combined by antipsychotic medication use from the 2005–2014 National Health and Nutrition Examination Survey (*n* = 16,960). (DOCX)

S2 Table Unadjusted and adjusted prevalence risk ratios (PRR), with 95% confidence intervals (CI), of the association between antipsychotic medication use, hepatitis core antibody and hepatitis C virus antibody (anti-HCV) among respondents to the 2005–2014 National Health and Nutrition Examination Survey (*n* = 12,722). (DOCX)
