# Supplementary figures and images for: Hepatitis B and C in individuals with a history of antipsychotic medication use: A population-based evaluation
Source: PLoS One. 2023 Apr 14;18(4):e0284323. doi: 10.1371/journal.pone.0284323 (PMC10104286; doi:10.1371/journal.pone.0284323)

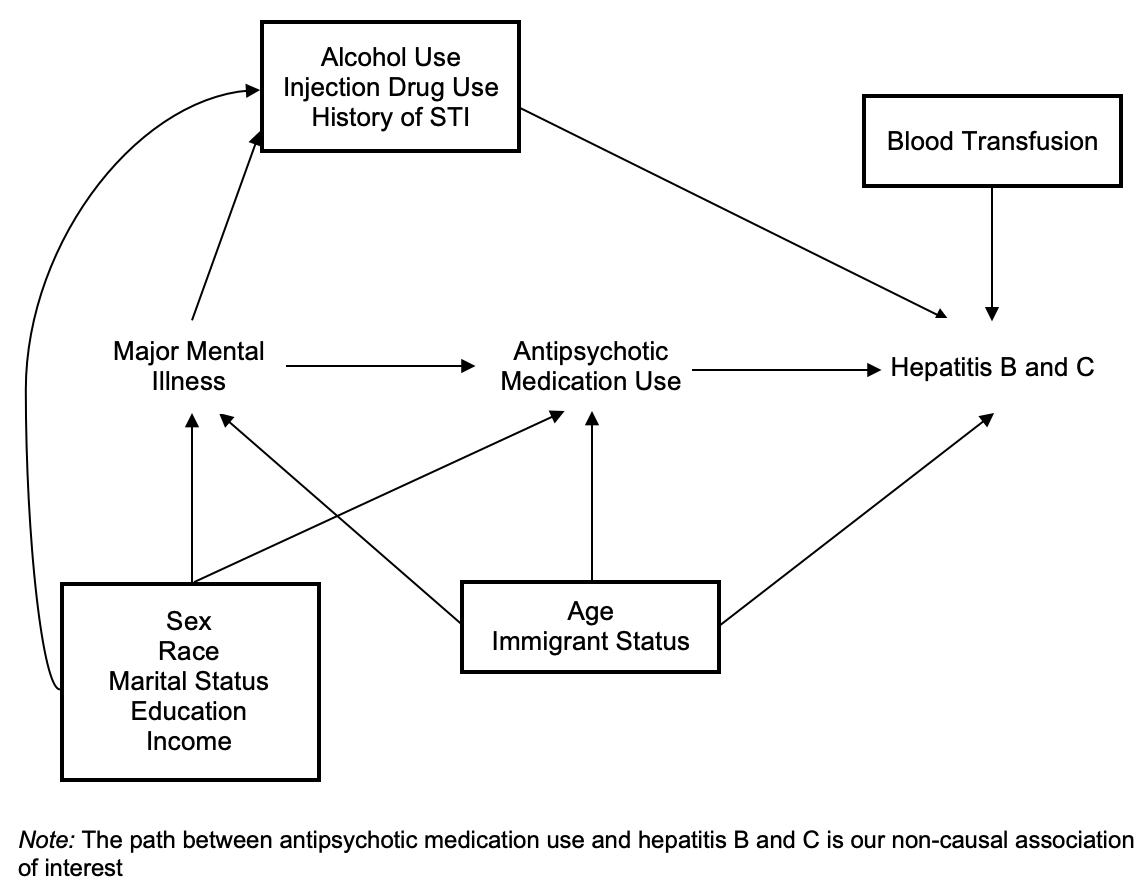

Supplement: S1 Fig — (TIF) [file pone.0284323.s001.tif]
