# Supplementary material for: Hepatitis B and C in individuals with a history of antipsychotic medication use: A population-based evaluation
Source: PLoS One. 2023 Apr 14;18(4):e0284323. doi: 10.1371/journal.pone.0284323 (PMC10104286; doi:10.1371/journal.pone.0284323)
Supplement: S1 Table — (DOCX) [file pone.0284323.s002.docx]

**S1 Table.** Distribution of hepatitis B core antibody and hepatitis C antibody combined by antipsychotic medication use from the 2005-2014 National Health and Nutrition Examination Survey (*n* = 16,960)

|  | Antipsychotic use  (*n* = 264) | | No Antipsychotic Use  (*n* = 16,696) | | *p^a^* |
| --- | --- | --- | --- | --- | --- |
|  | Wtd. % | (Unwtd. *n*) | Wtd. % | (Unwtd. *n*) |  |
| *HBV Core Antibody*  Positive  Negative | 7.0%  93.0% | (28)  (236) | 4.3%  95.7% | (963)  (15,733) | <0.05 |
| *HCV Antibody*  Positive  Negative | 6.7%  93.3% | (22)  (242) | 1.8%  98.2% | (332)  (16,364) | <0.001 |

*Notes:* Wtd. = weighted; Unwtd. = unweighted; *^a^*Pearson’s chi-square.
