# Supplementary material for: Hepatitis B and C in individuals with a history of antipsychotic medication use: A population-based evaluation
Source: PLoS One. 2023 Apr 14;18(4):e0284323. doi: 10.1371/journal.pone.0284323 (PMC10104286; doi:10.1371/journal.pone.0284323)
Supplement: S2 Table — (DOCX) [file pone.0284323.s003.docx]

**S2 Table.** Unadjusted and adjusted prevalence risk ratios (PRR), with 95% confidence intervals (CI), of the association between antipsychotic medication use, hepatitis core antibody and hepatitis C virus antibody (anti-HCV) among respondents to the 2005-2014 National Health and Nutrition Examination Survey (*n* = 12,722)

| Characteristic | Unadjusted | | | Adjusted | | |
| --- | --- | --- | --- | --- | --- | --- |
|  | PRR | [95% CI] | *p* | aPRR | [95% CI] | *p* |
| No HBV core antibody and no HCV antibody | | | | | | |
| HBV core antibody only | | | | | | |
| Antipsychotic Use  Yes  No | 1.19  1.00 | [0.57, 2.53]  Ref. | 0.63 | 0.93  1.00 | [0.43, 2.01]  Ref. | 0.84 |
| Age, *M* [95% CI] |  |  |  | 1.05 | [1.04, 1.06] | <0.001 |
| Sex, % (*n*)  Male  Female |  |  |  | 1.00  0.78 | Ref.  [0.62, 0.97] | <0.05 |
| Race, % (*n*)  Non-Hispanic White  Non-Hispanic Black  Hispanic  All other races |  |  |  | 1.00  4.90  0.83  6.10 | Ref.  [3.68, 6.53]  [0.50, 1.40]  [4.28, 8.70] | <0.001  0.48  <0.001 |
| Immigrant status, % (*n*)  Born in United States  Born outside United States |  |  |  | 1.00  3.33 | Ref.  [2.47, 4.49] | <0.001 |
| Marital status, % (*n*)  Married or cohabitating  Never married  Widowed, divorced, or separated |  |  |  | 1.00  1.21  1.11 | Ref.  [0.86, 1.71]  [0.77, 1.60] | 0.27  0.57 |
| Education, % (*n*)  College  High school graduate  Less than high school |  |  |  | 1.00  1.06  1.08 | Ref.  [0.74, 1.53]  [0.78, 1.49] | 0.74  0.65 |
| Income, *M* [95% CI] |  |  |  | 0.89 | [0.83, 0.96] | <0.01 |
| Blood transfusion, % (*n*)  Yes  No |  |  |  | 1.09  1.00 | [0.75, 1.60]  Ref. | 0.64 |
| Alcohol use, % (*n*)  Yes  No |  |  |  | 0.94  1.00 | [0.70, 1.26]  Ref. | 0.67 |
| Injection drug use, % (*n*)  Yes  No |  |  |  | 3.47  1.00 | [1.57, 7.67]  Ref. | <0.01 |
| Sexually transmitted infection, % (*n*)  Yes  No |  |  |  | 2.33  1.00 | [1.12, 4.85]  Ref. | <0.05 |
| HCV antibody only | | | | | | |
| Antipsychotic Use  Yes  No | 3.01  1.00 | [1.10, 8.24]  Ref. | <0.05 | 1.37  1.00 | [0.34, 5.51]  Ref. | 0.66 |
| Age, *M* [95% CI] |  |  |  | 1.08 | [1.06, 1.11] | <0.001 |
| Sex, % (*n*)  Male  Female |  |  |  | 1.00  0.70 | Ref.  [0.36, 1.33] | 0.27 |
| Race, % (*n*)  Non-Hispanic White  Non-Hispanic Black  Hispanic  All other races |  |  |  | 1.00  1.15  1.04  0.92 | Ref.  [0.71, 1.86]  [0.45, 2.41]  [0.31, 2.73] | 0.57  0.92  0.87 |
| Immigrant status, % (*n*)  Born in United States  Born outside United States |  |  |  | 1.00  0.46 | Ref.  [0.12, 1.70] | 0.24 |
| Marital status, % (*n*)  Married or cohabitating  Never married  Widowed, divorced, or separated |  |  |  | 1.00  0.95  0.85 | Ref.  [0.55, 1.64]  [0.47, 1.54] | 0.86  0.58 |
| Education, % (*n*)  College  High school graduate  Less than high school |  |  |  | 1.00  1.55  2.12 | Ref.  [0.75, 3.22]  [1.11, 4.05] | 0.24  <0.05 |
| Income, *M* [95% CI] |  |  |  | 0.72 | [0.59, 0.89] | <0.01 |
| Blood transfusion, % (*n*)  Yes  No |  |  |  | 1.86  1.00 | [1.04, 3.32]  Ref. | <0.05 |
| Alcohol use, % (*n*)  Yes  No |  |  |  | 1.58  1.00 | [0.94, 2.65]  Ref. | 0.08 |
| Injection drug use, % (*n*)  Yes  No |  |  |  | 33.93  1.00 | [18.82, 61.19]  Ref. | <0.001 |
| Sexually transmitted infection, % (*n*)  Yes  No |  |  |  | 0.00  1.00 | [0.00, 0.00]  Ref. | <0.001 |
| HBV core antibody and HCV antibody | | | | | | |
| Antipsychotic Use  Yes  No | 4.49  1.00 | [1.82, 11.08]  Ref. | <0.01 | 1.35  1.00 | [0.37, 4.93]  Ref. | 0.65 |
| Age, *M* [95% CI] |  |  |  | 1.16 | [1.13, 1.19] | <0.001 |
| Sex, % (*n*)  Male  Female |  |  |  | 1.00  0.49 | Ref.  [0.22, 1.08] | 0.08 |
| Race, % (*n*)  Non-Hispanic White  Non-Hispanic Black  Hispanic  All other races |  |  |  | 1.00  1.99  1.45  1.87 | Ref.  [1.04, 3.81]  [0.60, 3.49]  [0.68, 5.12] | <0.05  0.41  0.22 |
| Immigrant status, % (*n*)  Born in United States  Born outside United States |  |  |  | 1.00  0.46 | Ref.  [0.14, 1.46] | 0.18 |
| Marital status, % (*n*)  Married or cohabitating  Never married  Widowed, divorced, or separated |  |  |  | 1.00  1.61  0.94 | Ref.  [0.62, 4.22]  [0.41, 2.15] | 0.32  0.88 |
| Education, % (*n*)  College  High school graduate  Less than high school |  |  |  | 1.00  1.35  1.36 | Ref.  [0.58, 3.15]  [0.59, 3.14] | 0.49  0.47 |
| Income, *M* [95% CI] |  |  |  | 0.56 | [0.43, 0.72] | <0.001 |
| Blood transfusion, % (*n*)  Yes  No |  |  |  | 2.06  1.00 | [0.89, 4.78]  Ref. | 0.09 |
| Alcohol use, % (*n*)  Yes  No |  |  |  | 1.57  1.00 | [0.88, 2.81]  Ref. | 0.13 |
| Injection drug use, % (*n*)  Yes  No |  |  |  | 43.28  1.00 | [18.53, 101.10]  Ref. | <0.001 |
| Sexually transmitted infection, % (*n*)  Yes  No |  |  |  | 0.97  1.00 | [0.13, 7.27]  Ref. | 0.98 |

*Notes:* PRR = prevalence risk ratio; aPRR = multivariable prevalence risk ratio; Ref. = reference.
